# Collagen Nanofiber‐Lignin Composite Sponges with Adjustable Hierarchical Pore Structure for Efficient Low‐Frequency Sound Absorption

**DOI:** 10.1002/advs.202412583

**Published:** 2025-01-15

**Authors:** Yan Ma, Mu He, Jiaxuan Wang, Fuying Ma, Hongbo Yu, Yaxian Zhou, Shangxian Xie

**Affiliations:** ^1^ Department of Biotechnology College of Life Science and Technology Huazhong University of Science and Technology Wuhan 430074 China; ^2^ State Key Laboratory of Intelligent Manufacturing Equipment and Technology School of Mechanical Science and Engineering Huazhong University of Science and Technology Wuhan 430074 China; ^3^ Wuhan Second Ship Design and Research Institute Wuhan 430205 China; ^4^ Guangxi Shenguan Collagen Technology Research Institute Guangxi Shenguan Collagen Biological Group Wuzhou 543000 China

**Keywords:** collagen nanofibers, hierarchical pore structure, lignin, noise pollution

## Abstract

Current sound‐absorbing materials, reliant on nonrenewable resources, pose sustainability and disposal challenges. This study introduces a novel collagen‐lignin sponge (CLS), a renewable biomass‐based material that combines collagen's acoustic properties with lignin's structural benefits. CLSs demonstrate high porosity (>0.97), lightweight (10 mg cm^−^^3^), and exceptional broadband noise absorption performance (sound absorption coefficient exceeding 0.9 across 2000–6300 Hz). Due to their unique hierarchical and aligned pore structure, CLSs display superior low‐frequency sound‐absorbing capabilities and a high noise‐reduction coefficient of 0.64 (for a 30‐mm‐thick sample). A geometric model is also developed to evaluate and predict the sound absorption performance with high consistency to the experimental results. Additionally, the inclusion of lignin as a green crosslinker has significantly improved the thermal stability and compressive strength by ≈600% compared to collagen sponges alone. The innovative integration of collagen and lignin in this study not only leverages the benefits of renewable resources but also presents a cost‐effective and straightforward preparation process, positioning CLS as a promising alternative for the construction of sound‐absorbing materials seeking sustainable solutions.

## Graphical abstract

A facile and scalable collagen‐based nanofiber hierarchical porous sound‐absorbing sponge (CLS) is developed, inspired by the physiological functions of collagen nanofibers. The pore structure of CLSs can be effectively optimized by modulating lignin, which induces the entangled nanofiber network to transition into air cavities, thus forming a hierarchical porous architecture. Due to the unique hierarchical and aligned pore structure, CLSs display superior low‐frequency sound‐absorbing capabilities and lightweight properties.

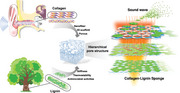

## 1 Introduction

With the rapid expansion of urbanization, noise pollution has emerged as a significant social issue, posing threats to the global economy, ecological environment, and human health.^[^ ^1^, ^2^ ^]^ An additional issue linked to urbanization is building‐related pollution, characterized by high levels of carbon emissions and waste generation. The construction sector accounts for ≈40% of global energy consumption and almost 38% of total annual CO_2_ emissions. Meanwhile, the construction industry contributes ≈40% of potable water pollution, 50% of landfill trash, and ≈23% of air pollution.^[^ ^3^ ^]^ Stringent regulations and increased awareness of noise pollution have driven the increased use of sound‐absorbing materials in the construction sector. Various sound‐absorbing materials, including petroleum‐based polymers (e.g., polyurethane foams and expanded/extruded polystyrene foams) and rock‐ and slag‐based fibers (e.g., mineral wool and glass wool), are extensively employed in building and construction sectors due to their superior sound absorption capabilities, thermal insulation, and enhanced fire‐retardant properties compared to natural fibers.^[^ ^4^, ^5^ ^]^ However, these synthetic materials, which depend on nonrenewable resources, generate pollutants and substantial carbon emissions during their manufacturing processes. Moreover, the poor biodegradability of synthetic materials, especially polymer foams, aggravates environmental challenges when disposed of as construction waste after use.^[^ ^6^, ^7^ ^]^ Replacing carbon‐intensive, nonrenewable, synthetic materials with renewable biomass‐based structures could reduce embodied carbon in buildings by 16%, making a significant environmental improvement.^[^ ^8^ ^]^ Therefore, developing advanced biomass‐based materials is crucial for mitigating architectural and environmental noise in the construction industry.

Despite advancements in sound‐absorbing materials,^[^ ^6^, ^8^, ^9^, ^10^, ^11^ ^]^ designing a renewable biomass‐based material with efficient sound absorption capacity, lightweight, high mechanical strength, and biodegradability remains a challenge. Evolution over millions of years has endowed nature with superior acoustic biosystems, such as the tympanic membrane in animals, which relies on collagen fibers for high‐frequency sound conduction.^[^ ^12^ ^]^ Additionally, exposure to randomly applied noise impulses has been shown to increase collagen in the heart, and low‐frequency noise increases cardiac collagens I and III in the extracellular matrix.^[^ ^13^, ^14^ ^]^ These physiological functions of collagen have inspired us to investigate its potential as a substrate for developing ultra‐high‐performance synthetic acoustic materials. Collagen, a natural nanofiber with a triple‐helix structure formed by three parallel polypeptide chains, has a rope‐like fiber structure of 280 nm in length and 1.4 nm in diameter (Figure S1a, Supporting Information).^[^ ^15^ ^]^ The nano‐scale diameter and high surface area of collagen fibers may facilitate the dissipation of sound energy through friction and viscous damping.^[^ ^16^, ^17^ ^]^ Moreover, the collagen sponge (CS) is a highly porous and interconnected structure,^[^ ^18^ ^]^ which may further increase the sound wave contact with the fiber surface and thus dissipate the sound energy suggesting that CS shall have the potential to be an ideal nanofibers sound‐absorbing material.^[^ ^19^ ^]^ Additionally, tanneries produce a significant amount of collagen waste. Converting this waste into value‐added products is both sustainable and economical. However, collagen sponges still lack the mechanical strength and thermostability required for industrial applications. Hence, there is an urgent need to develop a new method that improves the stability of collagen sponges and fabricates a controllable porous structure with superior sound absorption capacity.

Lignin, the most abundant renewable phenolic polymer on Earth, constitutes ≈15–40% of the dry weight in most terrestrial plants.^[^ ^20^ ^]^ Lignin is deposited in the cell wall during cell differentiation, essential for maintaining cell structural integrity and plant stiffness. It also protects against biological stresses by inhibiting enzymatic degradation of other components and exhibiting antibacterial activity against microorganisms.^[^ ^21^, ^22^ ^]^ Lignin features a complex network structure with methoxyl groups, phenolic hydroxyl groups, and terminal aldehyde groups within its polymer chains. The high density of active functional groups in lignin facilitates its modification and utilization, promoting compatibility with various polymers and enhancing composite material homogeneity.^[^ ^23^ ^]^ Thus, lignin, which is inexpensive and possesses numerous attractive properties including high thermal stability, biodegradability, antimicrobial activities, and favorable stiffness, serves as an ideal complementary modifier to overcome the structural disadvantages of CS.

Herein, a novel collagen‐lignin sponge (CLS) has been designed and fabricated from collagen and lignin by a robust and facile strategy. CLSs show a high porosity of >0.97, extraordinary noise‐reduction capability (with a sound absorption coefficient exceeding 0.9 across 2000–6300 Hz range), and a lightweight characteristic of 10 mg cm^−3^. Meanwhile, due to their unique hierarchical and aligned pore structure, CLSs display superior low‐frequency sound‐absorbing capabilities and achieve a high noise‐reduction coefficient of 0.64 for the 30 mm thick sample. Furthermore, the structural stability of CLSs has been greatly enhanced by the incorporation of lignin as a green crosslinker, leading to a significant increase in thermal stability and ≈600% improvement in compressive strength compared to collagen sponges. Additionally, numerical simulations for the sound absorption coefficient of the samples were conducted using different acoustic models. The simulation results demonstrated a similar trend to the experimental measurements, with the limp model also showing the predicted peak between 1500 and 2000 Hz. Although neither collagen nor lignin is a new biomaterial, the application of collagen as a sound‐absorbing material is an innovative idea. The exceptional sound absorption and lightweight properties of CLSs surpass those of existing natural biomass‐based sound‐absorbing materials and commercial sound absorbers. The source sustainability, cost‐effectiveness, and straightforward preparation process position CLS as a promising alternative for efficient sound‐absorbing materials. Our research introduces a groundbreaking advancement in the field of sustainable sound management with the development of CLSs. These novel biomaterials, distinguished by their exceptional acoustic performance and lightweight design, effectively transcend the limitations of current natural bio‐based materials. The innovative integration of collagen and lignin not only harnesses the inherent benefits of these renewable resources but also paves the way for a new generation of sound‐absorbing solutions. This bio‐inspired approach signifies a leap forward in material science, offering a sustainable and high‐performance alternative for the construction industry, and setting a new benchmark for eco‐friendly sound absorption technology.

## 2 Results and Discussion

### 2.1 Fabrication and Structure Characteristics of CLSs

**Figure 1:**
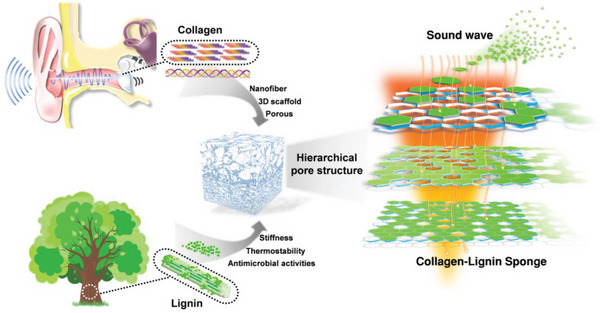
Schematic demonstrating the preparation and structure of the collagen‐lignin sponges (CLSs). A facile and scalable collagen‐based nanofiber hierarchical porous sound‐absorbing sponge was developed, which was inspired by the physiological functions of collagen nanofibers. The pore structure of CLSs can be effectively optimized by modulating lignin, which induces the entangled nanofiber network to transition into air cavities, thus forming a hierarchical porous architecture. Due to the unique hierarchical and aligned pore structure, CLSs display superior low‐frequency sound‐absorbing capabilities and lightweight properties.

**Figures** **1** and S2 (Supporting Information) display the preparation schematic diagram for the collagen‐lignin porous sponge. The fabrication process of CLSs involves four components: collagen, lignin, NaOH, and ultrapure water, and it can be divided into four main steps. Above all, collagen was dispersed in ultrapure water and stirred magnetically in an ice bath. Next, NaOH was added to quickly adjust the pH value to ≈9 by high‐speed stirring. The rapid adjustment of the collagen dispersion's pH to alkaline is essential for achieving material homogeneity. Selecting a pH of 9 is crucial for collagen, as the pH level significantly influences the solubility of collagen molecules, their interactions, and their molecular conformation. It has been reported that collagen solubility is pH‐dependent, peaking under basic conditions (≈95% at pH 9).^[^ ^24^ ^]^ Moreover, collagen films formed under alkaline conditions typically exhibit superior mechanical properties compared to those formed under acidic conditions.^[^ ^25^ ^]^ Li et al.^[^ ^26^ ^]^ also investigated in vitro collagen self‐assembly at a pH range of 6.0–10.5 at 30 °C, and summarized the diameter distributions of the fibrils with varying pH levels. The finding indicated that fibrillogenesis accelerated with increasing pH in this range, with collagen molecules forming fibrils of a constant diameter of ≈200 nm. From another perspective, in an alkaline environment, kraft lignin is solubilized by ionization of phenolic groups.^[^ ^27^ ^]^ Then, lignin powder was added to the collagen dispersion for further homogenization, and the solution was kept at room temperature in order to remove air bubbles.^[^ ^28^ ^]^ Subsequently, the samples were prechilled at 4 °C and then transferred to −80 °C for freezing and forming. Since the pore structure of the freeze‐dried sponges mirrors the ice‐crystal morphology after freezing, it is crucial to choose the appropriate freezing mode. Previous studies have demonstrated that the cooling rate determines the structural homogeneity while freezing temperature influences pore size during the freezing process. Notably, the pore size of the sponges decreases with lower as the freezing temperature.^[^ ^29^, ^30^, ^31^ ^]^ Finally, the obtained collagen‐lignin sponges were immersed in a coating solution consisting of polydimethylsiloxane (PDMS) to achieve CLSs with good hydrophobicity.

Considering the diversity of lignin and the scalability of CLSs preparation, three different types of industrial lignin, soluble lignin (SL), insoluble lignin (IL), and longlive lignin (LL), were selected and fabricated with collagen into CLS, named S‐CLS, I‐CLS and L‐CLS, respectively. The potential of these three CLSs for their acoustic and mechanical properties was investigated and compared. Ultimately, three types of CLSs were successfully obtained, all of which exhibited superior performance to a similar extent. The attenuated total reflectance Fourier transform infrared (ATR‐FTIR) spectra of CLSs were measured to characterize the interaction between lignin and collagen (Figure S3, Supporting Information). Apparent characteristic bands for the aromatic skeletal vibrations at 1600, 1506, and 1426 cm^−1^ were observed for the three types of CLSs.^[^ ^32^, ^33^ ^]^ The primary bands characteristic of collagen at 1630 cm^−1^ (amide I), 1548 cm^−1^ (amide II), 1238 cm^−1^ (amide III), and 1450 cm^−1^ (pyrrolidine rings of proline and hydroxyproline), as the region of the collagen fingerprint, could be clearly identified in CLSs.^[^ ^34^ ^]^ The absorption ratio of amide III to 1450 cm^−1^ (AIII/A1450), was ≈1, which indicated an intact triple helix structure of the collagen in the CLSs.^[^ ^35^ ^]^ The results suggested that the addition of lignin did not destroy the triple helix structure of collagen. In addition, we conducted an X‐ray diffraction (XRD) analysis of S‐CLSs to evaluate the influence of lignin on the crystal structure of collagen fibers (Figure S4 and Note S1, Supporting Information). It is evident that the triple helix structure of the collagen nanofiber is maintained in all S‐CLSs, inconsistent with FTIR results.^[^ ^36^ ^]^

### 2.2 Sound Absorption Properties of CLSs

**Figure 2:**
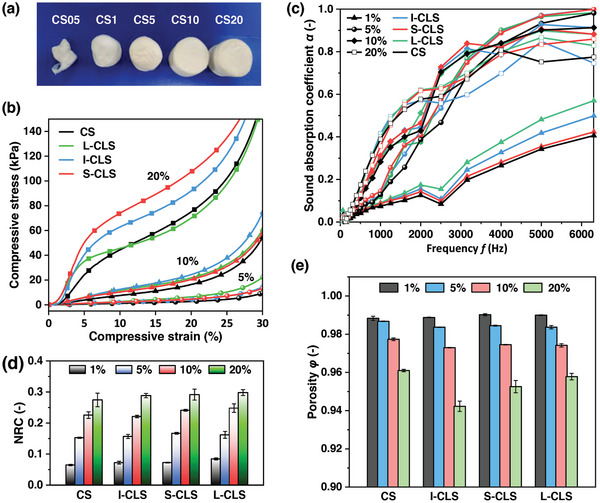
a) Optical images of the sponges for different collagen contents. b) Compressive stress‐strain curves of the sponges with different apparent densities. c) Sound absorption coefficients of the sponges with different apparent densities. d) Noise reduction coefficient (NRC) of the sponges with different apparent densities. e) Porosity of the sponges with different apparent densities (I‐CLS, insoluble lignin; S‐CLS, soluble lignin; L‐CLS, longlive lignin.

In order to investigate the impact of density on the frequency‐dependent acoustic absorption properties of the samples, collagen sponges with a thickness of 10 mm and densities of 1, 5, 10, 20, and 40 mg cm^−3^ were prepared. As demonstrated in **Figure** **2**a, CS05 and CS1 showed poor formability and structural stability, which could likely be attributed to their low density and loose fibrillar networks. As the density increased, an abundant population of collagen nanofibrils was highly interwoven in all directions, forming a network and assembling into scaffolds with high stability. The stability of various sponges could be deduced from their respective compressive stress‐strain curves, as depicted in Figure 2b. Additionally, Figure 2c,d illustrates the relationship between the noise absorption performance (thickness of 10 mm) of materials and their bulk density. The sample CS5 with low density possessed poor low‐frequency (<500 Hz) absorption and good high‐frequency (>500 Hz) absorption. This could be attributed to the constant friction and dissipation between high‐frequency sound waves and the macropore structure of CS5. Conversely, the high‐density CS20 displayed excellent low‐frequency absorption but poor high‐frequency absorption. This could be due to the fact that its small porous structure increased the dissipation probability of low‐frequency waves, while high‐frequency sound waves that featured lower penetration were reflected when entering CS20. As the bulk density increased, the probability of friction between sound waves and sponges also increased, consequently improving the noise absorption property, especially at low frequencies. However, when bulk density exceeded a certain threshold, the sponge's ability to resist sound energy entering also rose. This led to an increase in the reflection of the sound waves, thereby reducing the high‐frequency noise absorption property. For very low‐density fibrous materials, the fibers were spaced too far apart, which compromised the material's ability to attenuate acoustic energy. On the other hand, materials with very high densities suffered from high surface reflection and low acoustic penetration.^[^ ^37^ ^]^

Porosity is one of the major factors contributing to the sound absorption behavior of the material.^[^ ^17^ ^]^ The porous materials with high porosity and low density not only increase the reflection, refraction, and absorption of sound waves inside the material but can also have the characteristics of flexible assembly.^[^ ^38^ ^]^ Generally, the results showed that the porosity of porous materials ranged from 95% to 100%. The porosity of the material was negatively correlated with the density; as the density increased from 10 to 40 mg cm^−3^, the porosity decreased from 98.8% to 96.1% (Figure 2e). Therefore, CS5, with a density of 10 mg cm^−3^ and the highest porosity, was selected for the following study.

Thickness is the most direct parameter to reflect the noise absorption property of the sponges. CLSs with various thicknesses, ranging from 10 to 60 mm, were prepared to investigate how thickness affected the sound absorption performance of the materials (see details in Figure S5 and Note S2, Supporting Information). The results indicated that as the thickness increased, the sound absorption coefficient (SAC) of the sponge gradually increased and the absorption peak shifted toward lower frequencies. For the S‐CLSs (soluble lignin), the noise reduction coefficient (NRC) value increased significantly from 0.20 with a thickness of 10 mm to 0.74 with a thickness of 60 mm. Considering the available installation space,^[^ ^39^ ^]^ further research was conducted using a material thickness of 30 mm.

**Figure 3:**
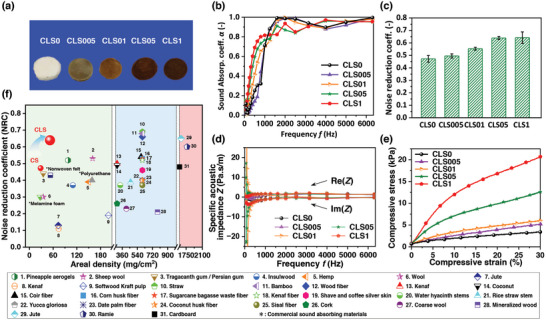
Effect of lignin content on the acoustic and mechanical properties. a) Optical images of the sponges with different lignin (soluble lignin, SL) contents. b) Sound absorption coefficient of CLSs with different SL contents. c) NRC of the sponges with different SL contents. d) Specific surface acoustic impedance of the sponges with different SL contents. e) Compressive stress‐strain curves with different SL contents. f) Comparison of the sound absorption properties of our sponges with other sound absorption materials.

Based on the fascinating effects of density and thickness on the sound absorption coefficient of CLSs, we have gained further insights into how lignin content affects the properties of sponges. The sponges were labeled as CLS0, CLS005, CLS01, CLS05, and CLS1, corresponding to 0%, 0.05%, 0.1%, 0.5% and 1% (w/v) lignin content calculated from the collagen dispersion. Except for special notes, all structural and performance characterizations were performed using CS5 with a density of 10 mg cm^−3^ as a sponge skeleton. As shown in **Figure** **3**a, the optical section of the lignin‐derived sponges revealed a gradual color deepening with varying lignin content from 0.05% to 1%. The impact of lignin content on the sound absorption performance of the CLSs is illustrated in Figure 3b. The trend was identical among the three types of lignin: with increasing lignin content, the SAC of the sponges gradually increased, and the absorption peak of the sponge shifted toward a lower frequency.

The NRCs of the S‐CLSs (soluble lignin) with increasing lignin loading amounts are 0.47, 0.50, 0.55, 0.64 and 0.64 (shown in Figure 3c), which are superior to those of L‐CLSs (longlive lignin) and I‐CLSs (insoluble lignin) (Figures S6 and S7, Supporting Information). We also compared the sound absorption properties of our sponges with other biobased sound‐absorbing materials in terms of NRC and areal density (Figure 3f and Table S1, Supporting Information), and found that the exceptional sound absorption and lightweight properties of CLSs surpassed those of existing natural biomass‐based sound absorption materials and commercial sound absorbers. Additionally, the corresponding surface impedance was calculated. The real (Re[*Z*]) and imaginary (Im[*Z*]) parts represent the transmission resistance and inertial acoustic resistance of sound waves, respectively (Figure 3d). The transmission and acoustic resistance of S‐CLSs vary with lignin content in the low‐frequency range (<1000 Hz), while remaining consistent with S‐CLS0 in the high‐frequency range (>1000 Hz).^[^ ^40^, ^41^ ^]^ Conversely, the addition of lignin results in a decrease in porosity from 98.8% in S‐CLS0 to 96.8% in S‐CLS1 (Figure S8a (Supporting Information). Apparently, this can be explained by the fact that lignin fills the empty voids of the nanofibers, thereby reducing their porosity. Based on the above acoustic parameters, the CLSs exhibit outstanding broadband sound absorption performance with an NRC up to 0.64, satisfying the requirement of high‐efficiency sound absorption materials (NRC ≥ 0.56).^[^ ^42^ ^]^ Meanwhile, the inclusion of lignin has effectively enhanced the sound absorption performance of the material.

The insulation properties against moisture are essential for the practical application of sound absorption materials.^[^ ^43^ ^]^ The water contact angle (WCA) on the surface of the S‐CLSs was investigated in Figure S9 and Note S3 (Supporting Information). By adopting the hydrophobic method of wrapping a layer of polydimethylsiloxane (PDMS) onto the surface of the CLSs, we obtained a hydrophobic sponge (HCLS) with a WCA of 120°,^[^ ^44^ ^]^ which endowed them with enhanced moisture insulation and maintained stable acoustic absorption properties. To assess the moisture resistance, we determined the weight retention rate by placing the HCLS in an ambient environment and weighing the mass at regular intervals. The sample weight remained remarkably consistent, with no discernible weight fluctuations (Figure S9b, Supporting Information), indicating that the sponge does not absorb water from the air. The moisture resistance property of HCLS was further demonstrated by moisture absorption and desorption tests in a high‐humidity environment (Figure S9c,d, Supporting Information). After exposing the HCLSs to water mist generated by a commercial humidifier for 12 h and conducting periodic weight measurements, we observed a 55% weight increase during high‐humidity exposure. Notably, when transferred to an ambient environment with 65% relative humidity, the adsorbed water in the samples was rapidly desorbed, allowing them to return to their original weight in ≈2.5 h (Figure S9e, Supporting Information). This demonstrates that the sponges possess good moisture resistance properties. Water primarily accumulates on the surfaces of the fibers and membranes due to their hydrophobic properties, resulting in rapid moisture desorption.

### 2.3 Characterization of Mechanical Performances of CLSs

The compressive mechanical properties of the CLSs are the primary performance parameters investigated in this study. The compressive stress‐strain curves of the different CLSs, namely CLS0, CLS005, CLS01, CLS05, and CLS1, are illustrated in Figure 3e, Figures S6d and S7d (Supporting Information). Observations from the study revealed that the addition of lignin resulted in a significant improvement in the compression properties of the CLSs, with the mechanical properties being positively correlated to lignin loading. Furthermore, S‐CLSs (soluble lignin) possess more preferable structural stability than other CLSs. At a compression strain of 30%, the compressive strengths of S‐CLS0, S‐CLS005, S‐CLS01, S‐CLS05, and S‐CLS1 are 3, 5, 6, 13, and 20 kPa, respectively. Compared to natural collagen sponges, the structural stability of CLSs has increased by ≈600% due to the involvement of lignin. In addition, thermogravimetric analysis (TGA) was used to characterize the thermal stability of CLSs, and the result indicates that the addition of lignin could significantly enhance the thermal stability of the porous sponges (Figure S10 and Note S4, Supporting Information). The introduction of lignin could have potentially endowed the material with thermal insulation and flame‐retardant properties to the material, as lignin is known to be a superior flame retardant (Figure S11, Supporting Information).^[^ ^45^ ^]^ The fire resistance was tested by burning with an alcohol lamp (≈500 °C). Compared to the collage The fire resistance properties of CLS were evaluated through exposure to an alcohol lamp, which simulates a high‐temperature environment of ≈500 °C. Compared to the collagen sponge (CS), the CLS exhibits significantly enhanced flame retardancy. Notably, the collagen sponge underwent rapid combusting within 8 s upon exposure to the flame (Figure S11a, Supporting Information). In contrast, CLS neither combusted nor dripped, and its temperature rapidly decreased upon withdrawal from the flame (Figure S11b, Supporting Information). These observations indicate CLS possesses commendable flame retardancy, self‐extinguishing, and antidripping performances. The infrared thermal imaging reveals the material's superior thermal insulation properties, as evidenced by the maintenance of room temperature at the surface of the material when subjected to a heating stage at 200 °C for a duration of 1 h (Figure S11c, Supporting Information).

Collagen has been extensively applied in biomaterials as an ideal matrix. However, the low mechanical strength and weak antimicrobial activities of unmodified collagen severely limit its applications.^[^ ^46^ ^]^ Multiple cross‐linking methods have been successfully applied to produce collagen‐based materials to improve their mechanics, thermal, and structural stability,^[^ ^18^ ^]^ including physical, biological, and chemical approaches. It is worth noting that both physical and biological methods, despite their superior cytocompatibility to chemical approaches, are often weaker than even the mildest chemical approach. Furthermore, the physical methods are associated with collagen denaturation. As such, the quest for an optimal collagen crosslinker continues.^[^ ^15^ ^]^ Here, lignin can be proposed as a new green cross‐linker, which not only greatly improves the stability of collagen sponges and strengthens their resistance to microbes, but also preserves the interconnected microstructure with high porosity.

When lignin was uniformly dispersed in collagen solution, collagen fibrils and lignin presumably formed networks via hydrogen bonding more likely or π−π stacking, thereby strengthening the cell walls and structure of collagen composite sponges. This result can be ascribed to the fact that the interior of the CLS was dense, which caused an increase in the number of force‐bearing points per unit area of the CLS. Consequently, the CLSs showed a significant increase in surface area, force‐bearing points, and total compressive force area.^[^ ^47^ ^]^ However, the excessive addition of lignin may have adverse effects on the continuity of the sponge's channels, ultimately leading to increased density, reduced porosity, and enhanced brittleness.^[^ ^38^ ^]^ This can be attributed to the agglomeration and nonuniform distribution of excess lignin, caused by strong π–π attraction and hydrogen bonding. Therefore, taking into account the above considerations, a suitable lignin content is essential to stabilize the CLS structure without compromising the sponge's acoustic properties and density.

### 2.4 The Microstructures and Morphology of CLSs

**Figure 4:**
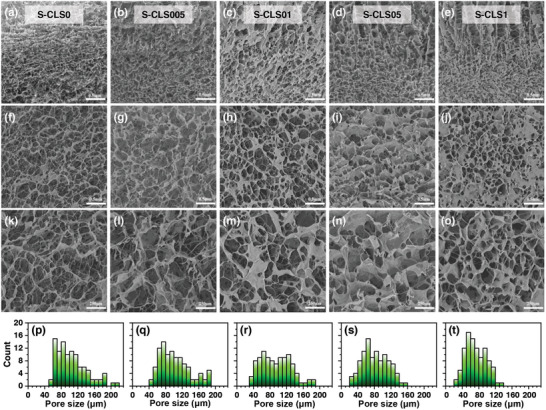
Demonstration of SEM images and pore size distribution of S‐CLSs (soluble lignin) with different lignin contents. a–e) SEM images along the longitudinal section. f–j) SEM images along the cross‐section. k–o) SEM images along the cross‐section with higher resolution. p–t) Pore size distribution of the cross‐section with higher resolution.

The morphology of the CLS cross‐section was captured through SEM with various lignin concentrations. The longitudinal‐sectional microstructures of CLSs are presented in **Figure** **4**a–e. The results revealed that the sponges containing lignin form a heterogeneous hierarchical structure with a loose upper layer and a dense lower layer, compared to the collagen sponge. Furthermore, this structure becomes even more apparent with increasing lignin content. As for the cross‐section depicted in Figure 4f–j, the hierarchical structures of CLSs consist of microporous structures and entangled networks, whereby the sound absorption behavior is governed by the interconnected porous structure of the materials and the thin wall surrounded by the nanofibrils. As lignin content increased, the entangled nanofiber networks gradually gave way to air cavity structures. This can contribute to the mechanical properties of CLSs and also explain their superiority over collagen sponges in terms of acoustic properties. The multiscale pore structure enhanced the number of air cavities connected inside fibrous porous materials, thus introducing additional mechanisms for sound absorption.^[^ ^48^ ^]^ In summary, lignin effectively optimized the pore structure of the CLSs; however, further increasing the lignin loading amount decreased the sound absorption performance. This occurred because excess lignin agglomerated and nonuniformly distributed due to the strong π–π interactions between aromatic rings and the formation of hydrogen bonds in abundant phenolic hydroxyl, carboxyl, methoxy, and other polar groups, leading to the reduction of connectivity of cell walls within CLSs.^[^ ^23^ ^]^

The pore size of the porous materials is one of the key factors affecting acoustic performance. Figure 4k–o displayed more details of the SEM image with a higher resolution, and the corresponding distributions of pore diameter were measured and recorded in Figure 4p–t. The measurements indicated that the mean pore size ranges from a few dozen microns to several hundred microns. The pore size of CLSs decreases as the lignin content increases to 1%, and the sponge exhibits a less porous structure.

The cross‐sectional SEM images of the stratified scanning of CLSs are presented in Figure S12 (Supporting Information), providing a more intuitive observation of the hierarchical pore structure of the composite sponges. Compared to S‐CLS0, the series S‐CLS05 exhibits a clear hierarchical pore structure: from the surface to the bottom, the material gradually becomes denser with the pore size decreasing and more concentrated in distribution with the number of micro‐cavities gradually increasing and the porosity decreasing (Figure S8b, Supporting Information). We ascribe the excellent sound absorption properties of CLSs to the following factors. First, the hierarchical structure results in the absorption of multiple acoustic waves between the layers, leading to a significant increase in the acoustic attenuation coefficient, thereby greatly enhancing the material's sound absorption capabilities. The hierarchical structure also increased the contact area of sound waves, providing more frictional resistance for the acoustic energy. To further characterize the acoustic performance of the hierarchical porous structure, the SACs of CLS were evaluated at normal (0°) and inverse (180°) incident angles (Figure S13, Supporting Information). The results demonstrated that both incident angles exhibited excellent sound absorption performance; notably, the absorption capability at 0° incidence was superior to that at 180°. This finding unveils the intrinsic advantages of the hierarchical porous structure in sound wave mitigation. Second, the nanofibers presented in CLSs possess a rough surface due to the presence of lignin. This property could increase the friction between sound waves and materials, leading to higher sound energy consumption during the progress of sound propagation. Third, a peak at a frequency of ≈1.5 kHz in the sound absorption curves can be observed, which could be attributed to fiber vibration. The resonant frequency of the CLSs at the absorption peak shows a tendency to shift to lower frequencies with increasing thickness, which could be attributed to the increased areal density of the sponges. Fiber vibration induced by sound waves would also play a crucial role in sound absorption. In addition, the presence of lignin particles in the sponges could cause resonating sound waves to generate heat dispersion and dissipate sound energy.

### 2.5 Numerical Simulation of Acoustic Properties of CLSs

#### 2.5.1 Geometric Modeling of Equivalent Structure via Image Processing

**Figure 5:**
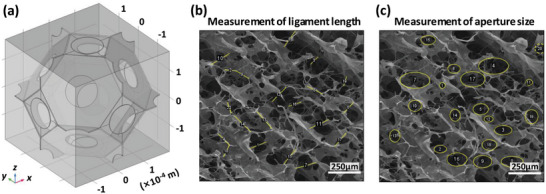
a) Geometric model of equivalent structure (truncated octahedron). b) Measurement of foam ligaments via ImageJ. c) Measurement of aperture size via ImageJ.

The geometric modeling and numerical simulation were conducted to predict the acoustic properties of CLSs in this study. As can be seen from the SEM images depicted in Figure 4, the sample exhibited a reticulated network. According to this morphology, a truncated octahedral (tetradecahedral) membranous foam can be used as the equivalent structure,^[^ ^49^ ^]^ which is a Kelvin cell shown in **Figure** **5**. The main geometric features include the length of the foam ligament *L*, the size of the aperture *r*, and the membrane thickness *t_m_*, of which the first two can be measured with ImageJ and then averaged as the geometric parameters of the equivalent structure, while the thickness *t_m_* can be inversely identified by using the measured porosity *φ*. According to this method, the equivalent geometric structures of S‐CLS05 with upper, middle, and lower layers were modeled in Comsol Multiphysics 5.6.

#### 2.5.2 Calculation of Transport Parameters by the Theory of Thermoviscous Acoustics

The correlation between microscopic geometry and macroscopic absorption properties can be established using the first‐principles calculations in the field of porous media acoustics.^[^ ^50^ ^]^ The theory considers the following aspects when sound waves enter the porous media: the dissipation due to viscous effects (more significant at low frequencies) or inertial effects (more significant at high frequencies), and the dissipation due to thermal effects (manifested as an isothermal process at low frequencies and adiabatic process at high frequencies). These effects are governed by a set of partial differential equations such as the Stokes equations, electric potential equations, and diffusion equations (combined with boundary conditions) and are termed “transport equations.” Physical quantities derived from the transport equations include several local quantities (velocity field *v*, pressure field *p*, electric potential *ψ*, excess temperature *T*) and eight transport parameters (porosity *φ*, viscous permeability *k*_0_, thermal permeability *k*_0_', viscous characteristic length *Λ*, thermal characteristic length *Λ*', viscous tortuosity *α*_0_, thermal tortuosity *α*_0_', high‐frequency tortuosity *α*_∞_). It is worth noting that the subsequently calculated absorption coefficients will be a function of these eight transport parameters. This relationship is summarized in **Table** **1**, and further details can be found in Note S5 (Supporting Information).

**Table 1:** Transport parameters and their governed equations.^[^ ^50^ ^]^

Transport parameters	Mechanism	Nomenclature	Transport equations	Postprocessing
*k*_0_,*α*_0_	Viscous effects at low frequencies	Stokes equations	$\left\{ \begin{array}{c} \eta \Delta u-\nabla p=-G,\\ \nabla \cdot u=0,\\ \mathrm{BC}:u=0\;\mathrm{at}\;\mathrm{interface},\\ \;u\;\mathrm{and}\;p\;\mathrm{are}\;\mathrm{periodic}. \end{array}\right.$	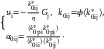
*α*_*∞*_,*Λ*	Inertial effects at high frequencies	Electric potential equations	$\left\{ \begin{array}{c} E=-\nabla \psi +e,\\ \nabla \cdot E=0,\;\\ \mathrm{BC}:E\cdot n=0\;\mathrm{at}\;\mathrm{interface},\\ \;\psi \;\mathrm{is}\;\mathrm{periodic}. \end{array}\right.$	$\left\{ \begin{array}{c} \;\alpha_{\infty }=\frac{\left\langle E^{2}\right\rangle }{{\left\langle E\right\rangle }^{2}}\;,\\ \Lambda =2\frac{\int_{\Omega_{f}}E^{2}dV}{\int_{\partial \Omega_{f}}E^{2}dS}. \end{array}\right.$
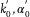	Isothermal process at low frequencies	Diffusion equations	$\left\{ \begin{array}{c} \Delta w=-1,\\ \mathrm{BC}:w=0\;\mathrm{at}\;\mathrm{interface},\\ \;w\;\;\mathrm{is}\;\mathrm{periodic}. \end{array}\right.$	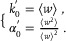
*φ*, *Λ′*	Adiabatic process at high frequencies	Purely geometric relationship	$\left\{ \begin{array}{c} \varphi =V_{f}/V,\\ \Lambda^{\prime }=2V_{f}/S, \end{array}\right.$	with *V_f_* the volume of solid phase, and *S* the area of solid‐fluid interface.

**Figure 6:**
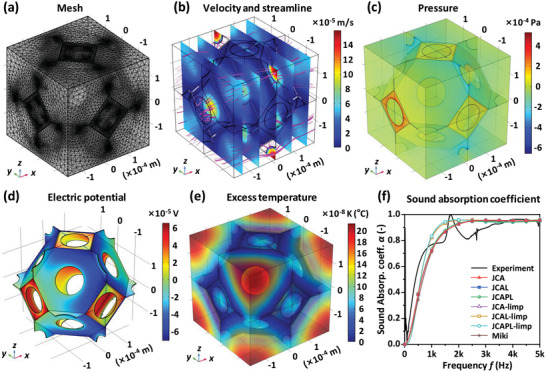
Numerical simulation of acoustic properties of CLSs. a) Finite element mesh for the equivalent structure. b) Illustration of velocity field and streamlines. c) Illustration of pressure field. d) Illustration of the electric potential field. e) Illustration of excess temperature field. f) Calculated sound absorption coefficient as a function of frequency using different acoustic models superimposed with experimental measurement.

Taking the upper layer of the CLS05 sample as an example, the transport parameters were simulated and obtained by the finite element method. The mesh for the equivalent structure is shown in **Figure** **6**a, and the microscopic local fields described in Table 1 are illustrated in Figure 6b–e, where Figure 6b,c demonstrate the velocity field *u* and the pressure field *p*, Figure 6d exhibits the potential field *ψ*, and Figure 6e depicts the excess temperature *T* (characterizing the diffusion field *w* in Table 1). The calculated viscous and thermal permeabilities are *k*_0_ = 443 µm^2^ and *k*_0_' = 2316 µm^2^, respectively; the viscous and thermal characteristic lengths are *Λ* = 68.4 µm and *Λ*' = 143 µm, respectively; and the calculated tortuosities are *α*_0_ = 1.98, *α*_0_' = 1.36 and *α*_∞_ = 1.35. It should be noted that different geometries will yield different transport parameters, in which the porosity *φ* and the permeability *k*_0_ have the dominant influence on the sound absorption, followed by the other parameters (*Λ*, *Λ*', *α*_∞_, *α*_0_, *α*_0_').

#### 2.5.3 Acoustic Propagation Models in Porous Media

As mentioned above, the sound absorption coefficient (at normal incidence) as a function of frequency can be derived from these transport parameters, which is called an “acoustic model.” Various acoustic models have been proposed by researchers based on different assumptions. They are mainly divided into analytical,^[^ ^51^, ^52^ ^]^ empirical,^[^ ^53^ ^]^ and semi‐phenomenological models,^[^ ^54^, ^55^, ^56^ ^]^ among which the most famous ones include the Miki model (belonging to the empirical model) and the JCA/JCAL/JCAPL series of models (belonging to the semi‐phenomenological model). The computational procedures of these models are listed in **Table** **2**, and their scope of application or range of frequencies have been widely studied.^[^ ^53^, ^54^, ^55^, ^56^ ^]^ It is worth mentioning that the table also includes a “limp” model, which is a modification based on the assumption of a flexible skeleton, whereas the usual case would assume a rigid skeleton for the solid phase. Therefore, the sound absorption coefficients under different acoustic models can be easily calculated from this theory.

**Table 2:** Acoustic models to predict sound absorption coefficient from transport parameters.

**Inputs**: the thickness of sample *L_s_*, constants of air, frequency *f*, transport parameters 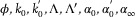 .
Constants of air (at 20 °C as example): *η* = 1.81 × 10^−5^ Pa · s, *ρ*_0_ = 1.205 kg/m^3^, *P*_*r*_ = 0.72, *c*_0_ = 343.4 m/s, *P*_0_ = 1.01 × 10^5^ Pa, *γ* = *c_p_* / *c_v_* = 1005/718 = 1.3997, *ν* = * η*/*ρ*_0_, *ν′* = ν/*P_r_*, *K_a_* = *γ* *P*_0_.
**Miki mode**l: only one parameter *k*_0_ will be used, satisfying *k*_0_ *f*/*η* < 0.01
Step 1: Calculate resistivity *σ* = *η*/*k*_0_, *Z_c_* = *ρ*_0_ *c*_0_[1 + 5.05(10^3^ *f*/*σ*)^−0.632^ − *i* · 8.43(10^3^ *f*/*σ*)^−0.632^], *k* = (*ω*/*c*_0_) · [1 + 7.81(10^3^ *f*/σ)^−0.618^ − *i* · 11.41(10^3^ *f*/*σ*)^−0.618^];
$\mathrm{Step}\;2:\;\mathrm{Calculate}\;Z=-iZ_{c}\cot\left(kL_{s}\right),\;\alpha =1-{|\frac{Z-\rho_{0}c_{0}}{Z+\rho_{0}c_{0}}|}^{2}.$
**JCA model**: five parameters *ϕ*, *k*_0_, *Λ*, *Λ′*, *α*_*∞*_ will be used; **JCAL model**: six parameters $\phi ,k_{0},\;k_{0}^{\prime },\;\Lambda ,\;\Lambda^{\prime },\;\alpha_{\infty }$ will be used; **JCAPL model**: eight parameters 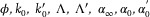 will be used:
Step 1: For JCA: *M* = 8*k*_0_ *α*_*∞*_/(*Λ* ^2^ *ϕ*), 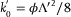 , *M*′ = *N* = *N*′ = 1; For JCAL: *M* = 8*k*_0_ *α*_*∞*_/*(Λ* ^*2*^ *ϕ*), $M^{\prime }=8k_{0}^{\prime }\alpha_{\infty }/\left({\Lambda^{\prime }}^{2}\phi \right)$, *N* = *N*′ = 1; For JCAPL: *M* = 8*k*_0_ *α*_*∞*_/(*Λ* ^2^ *ϕ*), $M^{\prime }=8k_{0}^{\prime }\alpha_{\infty }/\left({\Lambda^{\prime }}^{2}\phi \right)$, *N* = *M*/[4(*α*_0_/*α*_*∞*_ − 1)], $N^{\prime }=M/\left[4(\alpha_{0}^{\prime }-1)\right]$;
$\mathrm{Step}\;2:\;\mathrm{Calculate}\;\omega =2\pi f,\;\;\tilde{\omega }=\frac{\omega }{\nu }\;\cdot \frac{k_{0}\alpha_{\infty }}{\phi },\;\;{\tilde{\omega }}^{\prime }=\frac{\omega }{\nu^{\prime }}\;\cdot \frac{k_{0}^{\prime }}{\phi };$
$\mathrm{Step}\;3:\;\mathrm{Calculate}\;\;\tilde{f}=1-N+N\sqrt{1+\frac{M}{2N^{2}}i\tilde{\omega }},\;\;{\tilde{f}}^{\prime }=1-N^{\prime }+N^{\prime }\sqrt{1+\frac{M^{\prime }}{2{N^{\prime }}^{2}}i{\tilde{\omega }}^{\prime }},$ 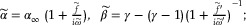
Step 4: If “**limp**” model: calculate *ρ*′ = *ρ*_1_ + *ϕρ*_0_ (*ρ*_1_: bulk density of the sample), 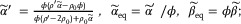 If **non** **‐** **limp** model: calculate ${\tilde{\alpha }}_{\mathrm{eq}}=\tilde{\alpha }\;/\phi ,\;\;{\tilde{\beta }}_{\mathrm{eq}}=\phi \tilde{\beta }$;
$\mathrm{Step}\;5:\;\mathrm{Calculate}\;\;{\tilde{q}}_{\mathrm{eq}}=\omega \sqrt{{\tilde{\alpha }}_{\mathrm{eq}}{\tilde{\beta }}_{\mathrm{eq}}\frac{\rho_{0}}{K_{a}}},\;\;{\tilde{Z}}_{\mathrm{eq}}=\sqrt{\frac{{\tilde{\alpha }}_{eq}}{{\tilde{\beta }}_{\mathrm{eq}}}\cdot \rho_{0}K_{a}}\;;$
$\mathrm{Step}\;6:\;\mathrm{Calculate}\;\;{\tilde{Z}}_{sn}=\frac{{\tilde{Z}}_{\mathrm{eq}}}{\rho_{0}c_{0}}\;\coth\left(i{\tilde{q}}_{eq}L_{s}\right),\;\alpha =1-{|\frac{{\tilde{Z}}_{sn}-1}{{\tilde{Z}}_{sn}+1}|}^{2}.$

#### 2.5.4 Prediction of Sound Absorption Coefficient Using Transfer Matrix Approach

Meanwhile, the studied samples can be considered as multi‐layers connected in series. According to the above method, the equivalent properties of the upper, middle, and lower layers of the structure are calculated individually, and then the transfer matrix approach will be used to obtain the overall absorption curve.^[^ ^57^ ^]^ The final sound absorption coefficient *α_n_* of the multilayer material can be determined by the following formula:

(1) $$T_{i}=\left[\begin{array}{cc} \cos\left(k_{c}h\right) & jZ_{p}\sin\left(k_{c}h\right)\\ j\sin\left(k_{c}h\right)/Z_{p} & \cos\left(k_{c}h\right) \end{array}\right]$$

(2) $$T_{\mathrm{total}}=\prod_{i=1}^{n}T_{i}=\left[\begin{array}{cc} T_{11} & T_{12}\\ T_{21} & T_{22} \end{array}\right]$$

(3) $$Z_{s}=T_{11}/T_{21}$$

(4) αn=4ReZs/ρ0c01+ReZs/ρ0c02+ImZs/ρ0c02

where $k_{c}=\omega \sqrt{\rho_{\mathrm{eff}}/K_{\mathrm{eff}}}$ is the complex wavenumber of the porous material, $Z_{p}=\sqrt{\rho_{\mathrm{eff}}\cdot K_{\mathrm{eff}}}$ is the acoustic impedance, *h* is the thickness, ρ_0_ is the density of air and *c*_0_ is the celerity of sound. Accordingly, the numerical simulation for the total acoustic absorption coefficient of the sample (CSL05) is obtained, as illustrated in Figure 6f, which is also superimposed on the experimental curve. It can be observed that the numerical simulation results are consistent with the experimental measurement, and the sound absorption curves predicted by the different models have the same trend with the experimental curve, among which the limp model has a better agreement at low frequencies and its predicted peak also appears between 1500 and 2000 Hz.

The discrepancies between the experimental results and the simulated outcomes may be ascribed to the foundational assumptions regarding the solid medium with acoustical models. Specifically, these models posit the collagen‐lignin network as either perfectly rigid or entirely limp; whereas experimental observations indicate that the solid network exhibits elastic behavior. This elasticity engenders localized resonances, which in turn augment the dissipation of sound energy and reduce the sound reflection. In addition, it is imperative to acknowledge the inapplicability of experimental data at frequencies ≈0 Hz (absorption coefficient ≈0.9). This is due to operational constraints of the impedance tube we used (type 2716C, Brüel & Kjær, Denmark), which specifies a measurable frequency range of 63–1600 Hz for a tube diameter of 100 mm. Therefore, experimental data below 63 Hz are deemed unreliable, as the true absorption coefficient is usually negligible at such low frequencies. This observation aligns with the findings from our simulations.

In addition, to further investigate the potential of CLS as an omnidirectional sound absorber, we conducted numerical simulations using established methodological procedures to predict oblique incident absorption performance. The simulation curves derived from the JCAPL‐limp model at various oblique incidences (from 60° to −60°) showed consistent sound absorption with that observed at normal incidence (Figure S14, Supporting Information). This indicates the promising potential of CLS for omni‐directional sound absorption. Moreover, the numerical simulations for predicting oblique incident absorption performance provide valuable insights for designing an omni‐directional sound absorber. Therefore, geometric modeling and parametric optimization of existing materials through theoretical analysis and numerical simulations could be a better way to evaluate the final sound absorption performance, thus eliminating the costly and tedious experimental manipulations. This strategy has great potential for the design of new hybrid materials.

## 3 Conclusion

In summary, we have developed a facile and scalable collagen‐based nanofiber hierarchical porous sponge through a simple blending of collagen and lignin, followed by a freeze‐drying process. The CLSs exhibit characteristics such as substantial resource availability, cost‐effectiveness, and ease of preparation, making them suitable for large‐scale production. Taking advantage of the nanofiber structure, CLSs show a high porosity of >0.97, extraordinary noise‐reduction capability (sound absorption coefficient over 0.9 in 2000–6300 Hz), and ultralight properties of 10 mg cm^−3^. The incorporation of lignin has effectively optimized the pore structure of CLSs, suggesting that the entangled nanofiber network transitions into an air cavity structure, forming a hierarchical porous architecture. Due to their unique hierarchical and aligned pore structure, CLSs exhibit superior low‐frequency sound absorption capabilities, achieving a high noise‐reduction coefficient of 0.64 for the 30 mm thick sample. Moreover, the structural stability of CLSs has been greatly enhanced by the incorporation of lignin as a green crosslinker, leading to a significant increase in thermal stability and approximately a 600% enhancement in compression strength compared to natural collagen sponges. Additionally, lignin may confer thermal insulation and flame‐retardant properties to our material. Numerical simulations for the sound absorption coefficient of the samples were also conducted using different acoustic models. The simulation results show a similar trend to the experimental measurements, with the limp model also indicating the predicted peak between 1500–2000 Hz.

The material is unique in its combination of renewable source materials, high porosity, lightweight properties, high sound absorption, high mechanical robustness, thermal insulation, and flame retardant, as well as in its efficient, cost‐effective, and scalable manufacturing. These attributes make CLSs a promising option as a sustainable construction material for improved noise regulation. We anticipate that the development of CLSs, with their superior acoustic performance and lightweight properties, not only overcomes the inherent bottleneck of existing natural bio‐based materials but also provides bioinspired approaches for the design and manufacturing of high‐performance sound absorption materials.

## 4 Experimental Section

### Materials and Chemicals

Bovine type I collagen (molecular weight = 410 kDa) was provided by Guangxi Shenguan Collagen Biological Group Co., Ltd. (Guangxi, China), with an approximate water content of 88%. The structure and molecular weight of the collagen were characterized by SEM and SDS‐PAGE analysis (Figure S1, Supporting Information). Two types of alkali lignin with different elemental compositions, termed herein as SL (low sulfonate content, cata # 471003, soluble in water) and IL (cata # 370959, insoluble in water), were purchased from Sigma‐Aldrich (Shanghai, China). Longlive lignin (enzymolysis lignin), denoted as LL in this work, was supplied via Shandong Longlive Bio‐technology Co., Ltd (Shandong, China). All other chemicals of analytical grade used in this study were purchased from Shanghai Aladdin Biochemical Technology Co., Ltd. (Shanghai, China). Ultrapure water was used in all experiments.

### Fabrication of Collagen‐Lignin Sponges

In the typical process for preparing collagen‐lignin sponges (CLSs), 5 g of collagen was dispersed in 100 mL of ultrapure water and stirred magnetically in an ice bath for 24 h to acquire a 5 wt.% collagen dispersion. Then, 1 m NaOH was used to promptly adjust the pH to ≈9 under constant stirring until the value stabilized. Later, lignin powder was introduced to the collagen dispersion for additional homogenization. After being stirred for 12 h, the mixture solutions were poured into a preprepared mold and left at ambient temperature to remove air bubbles. Following this, the samples were prechilled at 4 °C before being transferred to −80 °C for freezing and formation. The frozen block was then moved to a lyophilizer, where it underwent vacuum freeze‐drying for 48 h to ultimately obtain the sponges. CLSs with different lignin species, soluble lignin, insoluble lignin, and longlive lignin were fabricated, and the sponges were labeled as S‐CLS, I‐CLS, and L‐CLS, respectively, in accordance with the added lignin species. Throughout the preparation process for the sponges, the ambient temperature and humidity were carefully maintained at 25 °C ± 2 °C and 45% ± 5%, respectively. Additionally, various CS with densities ranging from 1 to 40 mg cm^−3^ were obtained by adjusting the concentration of collagen dispersions to 0.5%, 1%, 5%, 10%, and 20%. The sponges were named CS05, CS1, CS5, CS10, and CS20 respectively. To prepare CLSs with different lignin contents, 0.05, 0.1, 0.5, and 1 g of lignin were dissolved in 100 mL of collagen dispersion, resulting in lignin contents ranging from 0.05 to 1%. The sponges were labeled as CLS0, CLS005, CLS01, CLS05, and CLS1, corresponding to 0%, 0.05%, 0.1%, 0.5%, and 1% (w/v) lignin content. To achieve hydrophobicity, the sponges were submerged in a coating solution containing 1 g of polydimethylsiloxane (PDMS) with 10% curing agent dispersed in 30 mL of N‐hexane, resulting in the production of hydrophobic CLSs.

### Characterization and Measurement

The density of the CLS was calculated using the measured mass and geometry of each sample. The morphology and microstructures (including pore size) of the sponges were characterized using scanning electron microscopy (SEM) (Quanta 200 FEG, FEI, Netherlands). Porosity was determined via a simple liquid displacement method,^[^ ^38^ ^]^ whereby each sample was weighed (*m*_0_) and completely immersed in ethanol in a container, then weighed together (*m*_1_). The container was placed in a vacuum desiccator and vacuumed until no air bubbles escaped from the sponge. The sample was then removed, and the container with residual ethanol was weighed again (*m*_2_). The measurements were performed three times in parallel. The porosity (*φ*) of the CLS sample was then calculated according to the following equation: Porosity *φ* = $\frac{m_{1}-m_{2}-m_{0}}{m_{1}-m_{0}}\times 100\%$. The moisture resistance property of the sponges was characterized by the following method: placing them in an ambient environment for 49 days, with 90% ± 5% humidity and a temperature of 25 °C ± 2 °C, measuring their weight changes. Additionally, the sponges were placed at the outlet of a commercial humidifier for moisture absorption and then kept in an atmosphere with a relative humidity of ≈65% ± 5% and a temperature of 25 °C ± 2 °C for moisture desorption. The water content (*w*) in the sponges was calculated using the following equation: 𝑤 (%) = $\frac{m_{2}-m_{1}}{m_{1}}\times 100\%$, where *m_1_* is the initial weight of the samples, and *m_2_* is the weight of the samples after moisture absorption.

The ATR‐FTIR spectroscopic analysis was performed using a Nicolet iS10 Spectrometer (Vertex 70, Bruker, Germany). The XRD patterns from the samples were recorded with an X‐ray diffractometer (SmartLab‐SE, Rigaku, Japan) equipped with Cu Kα radiation with 2*θ* ranging from 5° to 55° at an 8° min^−1^ scanning rate. Mechanical compression tests were conducted using a universal testing machine (CMT4104, Xinsansi, China) with a 10 kN load cell. The loading and unloading rates were set to 10 mm min^−1^ during the stress‐strain tests. Three replicates were measured for each type of specimen. The thermogravimetric‐differential thermal analyzer (TG‐DTA) (Pyres Diamond TG/DTA PerkinElmer, USA) was utilized to measure thermal stability at temperatures ranging from 35 to 800 °C, under a nitrogen atmosphere and heating rate of 20 °C min^−1^. The fire resistance was tested by burning with a butane lighter. The water contact angle (WCA) was detected by a contact angle goniometer (OCA20, DataPhysics, Germany), with a controlled water droplet volume of 2 µL. Six positions were measured for each material to obtain an experiment plot, which represents the average. The plotted error bars indicate the experimental standard deviation from the mean.

The sound absorption coefficient (SAC) of the sponges obtained was evaluated using an impedance tube (Type 2716C, Brüel & Kjær, Denmark). The frequency ranges of 63–1600 Hz and 1000–6300 Hz were measured using a large tube (diameter of 100 mm) and a small tube (diameter of 30 mm), respectively. Cylindrical CLSs with diameters of 100 and 30 mm were prepared for measurement. The noise absorption test was conducted in the range of 63–6300 Hz based on relevant test methods and research significance, and the results were plotted into absorption coefficient curves. Three replicates were measured for each type of specimen, and each sample was tested three times to minimize the error. All sound absorption coefficient tests were performed at a temperature of 25 ± 2 °C and humidity of 45 ± 5%. The noise reduction coefficient (NRC) is the arithmetic mean value of the sound absorption coefficient of CLSs at 250, 500, 1000, and 2000 Hz. The presented values are the average values calculated from three measurements.

## Conflict of Interest

The authors declare no conflict of interest.

## Author Contributions

Y.M. and M.H. contributed equally to this work. S.X., Y.M., Y.Z., and H.Y. conceived the idea and designed the experiments. Y.M. contributed to the CLSs fabrication and characterization. J.W. and Y.M. contributed to the sound‐absorption measurement. M.H. contributed to the sound‐absorption simulations. S.X. and Y.Z. provided useful suggestions for raw materials selection and CLSs fabrication. S.X., Y.M. and M.H. collaboratively analyzed the data. Y.M. and M.H. drafted the manuscript. X.S., Y.Z., H.Y. and F.M. revised the manuscript. All authors reviewed and commented on the paper.

## Supporting information



Supporting information

## Data Availability

The data that support the findings of this study are available from the corresponding author upon reasonable request.

## References

[1] T. Munzel , M. Sorensen , A. Daiber , Nat. Rev. Cardiol. 2021, 18, 619. 33790462 10.1038/s41569-021-00532-5

[2] M. Sorensen , G. Pershagen , Eur. Heart J. 2019, 40, 604. 30496398 10.1093/eurheartj/ehy768

[3] J. Min , G. Yan , A. M. Abed , S. Elattar , M. Amine Khadimallah , A. Jan , H. Elhosiny Ali , Fuel 2022, 326, 124842.

[4] S. Schiavoni , F. D׳Alessandro , F. Bianchi , F. Asdrubali , Renew. Sust. Energ. Rev. 2016, 62, 988.

[5] T. Yang , L. Hu , X. Xiong , M. Petrů , M. T. Noman , R. Mishra , J. Militký , Sustainability‐Basel 2020, 12, 8477.

[6] M. Fattahi , E. Taban , P. Soltani , U. Berardi , A. Khavanin , V. Zaroushani , J. Build Eng. 2023, 77, 107468.

[7] C. Hill , A. Norton , J. Dibdiakova , Energy Build. 2018, 162, 12.

[8] X. Zhao , Y. Liu , L. Zhao , A. Yazdkhasti , Y. Mao , A. P. Siciliano , J. Dai , S. Jing , H. Xie , Z. Li , S. He , B. C. Clifford , J. Li , G. S. Chen , E. Q. Wang , A. Desjarlais , D. Saloni , M. Yu , J. Kośny , J. Y. Zhu , A. Gong , L. Hu , Nat. Sustain. 2023, 6, 306.

[9] M. Olivares‐Marín , S. Román , V. Gómez Escobar , C. Moreno González , A. Chaves‐Zapata , B. Ledesma , J. Clean Prod. 2023, 425, 138903.

[10] Z.‐J. Nie , J.‐X. Wang , C.‐Y. Huang , J.‐F. Feng , S.‐T. Fan , M. Tan , C. Yang , B.‐J. Li , S. Zhang , Chem. Eng. J. 2022, 446.

[11] S. Mehrzad , E. Taban , P. Soltani , S. E. Samaei , A. Khavanin , Build. Environ. 2022, 211, 108753.

[12] K. N. O'Connor , M. Tam , N. H. Blevins , S. Puria , Laryngoscope 2009, 118, 483. 10.1097/MLG.0b013e31815b0d9f 18091335

[13] E. Antunes , G. Borrecho , P. Oliveira , A. P. A. Matos , J. Brito , A. Águas , S. JMd , Int. J. Clin. Exp. Pathol. 2013, 6, 2333. 24228094 PMC3816801

[14] H. Ising , H.‐J. Marker , T. Gunther , H. Gelderblom , M. Ozel , Environ. Int. 1979, 2, 95.

[15] A. Sorushanova , L. M. Delgado , Z. Wu , N. Shologu , A. Kshirsagar , R. Raghunath , A. M. Mullen , Y. Bayon , A. Pandit , M. Raghunath , D. I. Zeugolis , Adv. Mater. 2019, 31. 10.1002/adma.201801651 30126066

[16] B. Gao , L. Zuo , B. Zuo , Fibers Polym. 2016, 17, 1090.

[17] S. Selvaraj , S. Ramalingam , S. Parida , J. R. Rao , N. F. Nishter , J. Hazard. Mater. 2021, 405, 124231. 33129600 10.1016/j.jhazmat.2020.124231

[18] L. Sun , B. Li , D. Yao , W. Song , H. Hou , J. Mech. Behav. Biomed. Mater. 2018, 80, 51. 29414475 10.1016/j.jmbbm.2018.01.006

[19] X. Li , Y. Peng , Y. He , C. Zhang , D. Zhang , Y. Liu , Nanomaterials 2022, 12, 1123.35407241

[20] A. J. Ragauskas , G. T. Beckham , M. J. Biddy , R. Chandra , F. Chen , M. F. Davis , B. H. Davison , R. A. Dixon , P. Gilna , M. Keller , P. Langan , A. K. Naskar , J. N. Saddler , T. J. Tschaplinski , G. A. Tuskan , C. E. Wyman , Science 2014, 344, 1246843. 24833396 10.1126/science.1246843

[21] L. Jones , A. R. Ennos , S. R. Turner , Plant J. 2001, 26, 205. 11389761 10.1046/j.1365-313x.2001.01021.x

[22] D. Kai , M. J. Tan , P. L. Chee , Y. K. Chua , Y. L. Yap , X. J. Loh , Green Chem. 2016, 18, 1175.

[23] Y. Li , F. Li , Y. Yang , B. Ge , F. Meng , J. Polym. Eng. 2021, 41, 245.

[24] V. Perez‐Puyana , A. Romero , A. Guerrero , J. Biomed. Mater. Res. A 2016, 104, 1462. 26833811 10.1002/jbm.a.35671

[25] T. Zhang , Z. Yu , Y. Ma , B.‐S. Chiou , F. Liu , F. Zhong , Food Hydrocoll. 2022, 124, 107270.

[26] Y. Li , A. Asadi , M. R. Monroe , E. P. Douglas , Mater. Sci. Eng. C 2009, 29, 1643.

[27] R. Liu , L. Dai , C. Xu , K. Wang , C. Zheng , C. Si , ChemSusChem 2020, 13, 4266. 32462781 10.1002/cssc.202000783

[28] Y.‐C. Nho , Y.‐M. Lim , H.‐J. Gwon , E.‐K. Choi , Korean J. Chem. Eng. 2010, 26, 1675.

[29] M. G. Haugh , C. M. Murphy , F. J. O'Brien , Tissue Eng. Part C Methods 2010, 16, 887. 19903089 10.1089/ten.TEC.2009.0422

[30] F. J. O'Brien , B. A. Harley , M. A. Waller , I. V. Yannas , L. J. Gibson , P. J. Prendergast , Technol. Health Care 2007, 15, 3. 17264409

[31] F. J. O'Brien , B. A. Harley , I. V. Yannas , L. J. Gibson , Biomaterials 2005, 26, 433. 15275817 10.1016/j.biomaterials.2004.02.052

[32] J. Zhao , W. Xiuwen , J. Hu , Q. Liu , D. Shen , R. Xiao , Polym. Degrad. Stab. 2014, 108, 133.

[33] X. Wang , H. Ren , Appl. Surf. Sci. 2008, 254, 7029.

[34] Z. S. Silva Junior , S. B. Botta , P. A. Ana , C. M. Franca , K. P. Fernandes , R. A. Mesquita‐Ferrari , A. Deana , S. K. Bussadori , Sci. Rep. 2015, 5, 11448. 26101184 10.1038/srep11448 PMC4477230

[35] M. T. Arafat , G. Tronci , J. Yin , D. J. Wood , S. J. Russell , Polymer 2015, 77, 102.

[36] M.‐M. Giraud‐Guille , L. Besseau , C. Chopin , P. Durand , D. Herbage , Biomaterials 2000, 21, 899. 10735466 10.1016/s0142-9612(99)00244-6

[37] R. Dunne , D. Desai , R. Sadiku , Acoust. Aust. 2017, 45, 453.

[38] H. Pu , X. Ding , H. Chen , R. Dai , Z. Shan , Environ. Technol. Innov. 2021, 24, 101874.

[39] J. Zhu , J. Sun , H. Tang , J. Wang , Q. Ao , T. Bao , W. Song , Powder Technol. 2016, 301, 1235.

[40] W. He , M. Liu , X. Peng , F. Xin , T. J. Lu , Phys. Fluids 2021, 33, 063606.

[41] D. Zong , L. Cao , X. Yin , Y. Si , S. Zhang , J. Yu , B. Ding , Nat. Commun. 2021, 12, 6599. 34782622 10.1038/s41467-021-26890-9 PMC8593031

[42] B. T. Samuel , M. Barburski , E. Witczak , I. Jasinska , Materials 2021, 14, 6220. 34683811 10.3390/ma14206220 PMC8539478

[43] M. J. Nine , M. Ayub , A. C. Zander , D. N. H. Tran , B. S. Cazzolato , D. Losic , Adv. Funct. Mater. 2017, 27, 1703820.

[44] Y.‐Q. Zhang , Y.‐H. Jiang , Y.‐N. Qin , Q.‐D. An , L.‐P. Xiao , Z.‐H. Wang , Z.‐Y. Xiao , S.‐R. Zhai , Coll. Surf A Physicochem. Eng. Asp. 2022, 643, 128790.

[45] M. A. R. Bhuiyan , A. Ali , H. Akter , M. A. R. Dayan , M. J. Hossen , M. J. Abden , A. N. Khan , Appl. Energy 2023, 345, 121317.

[46] L. Sun , B. Li , D. Jiang , H. Hou , Colloids Surf B 2017, 159, 89. 10.1016/j.colsurfb.2017.07.061 28780464

[47] J. A. Belgodere , S. A. Zamin , R. M. Kalinoski , C. E. Astete , J. C. Penrod , K. M. Hamel , B. C. Lynn , J. S. Rudra , J. Shi , J. P. Jung , ACS Appl. Bio Mater. 2019, 2, 3562. 10.1021/acsabm.9b00444 35030742

[48] J. Cucharero , S. Ceccherini , T. Maloney , T. Lokki , T. Hänninen , Cellulose 2021, 28, 4267.

[49] M. Tan Hoang , C. Perrot , J. Appl. Phys. 2012, 112, 054911.

[50] C. Perrot , F. Chevillotte , M. Tan Hoang , G. Bonnet , F.‐X. Bécot , L. Gautron , A. Duval , J. Appl. Phys. 2012, 111, 014911.

[51] M. A. Biot , J. Acoust. Soc. Am. 1956, 28, 168.

[52] M. A. Biot , J Acoust. Soc. Am. 1956, 28, 179.

[53] Y. Miki , J. Acoust. Soc. Jan (E) 1990, 11, 25.

[54] Y. Champoux , J.‐F. Allard , J. Appl. Phys. 1975, 70, 1991.

[55] S. R. Pride , F. D. Morgan , A. F. Gangi , Phys. Rev. B 1993, 47, 4964. 10.1103/physrevb.47.4964 10006656

[56] D. Lafarge , *Doctoral Thesis*, Univeristy of Le Mans 1993.

[57] Z. Liu , J. Zhan , M. Fard , J. L. Davy , Appl. Acoust. 2017, 121, 25.

