# Supplementary material for: Collagen Nanofiber‐Lignin Composite Sponges with Adjustable Hierarchical Pore Structure for Efficient Low‐Frequency Sound Absorption
Source: Adv Sci (Weinh). 2025 Jan 15;12(10):2412583. doi: 10.1002/advs.202412583 (PMC11904960; doi:10.1002/advs.202412583)
Supplement: Supplementary file 1 — Supporting information [file ADVS-12-2412583-s001.docx]

**Collagen nanofiber-lignin composite sponges with adjustable hierarchical pore structure for efficient low-frequency sound absorption**

*Yan Ma^#^, Mu He^#^, Jiaxuan Wang, Fuying Ma, Hongbo Yu^*^, Yaxian Zhou^*^, Shangxian Xie^*^*

Y. Ma, F. Ma, H. Yu, S. Xie

Department of Biotechnology, College of Life Science and Technology, Huazhong University of Science and Technology, Wuhan, 430074, China

Correspondence to: shangxian_xie@hust.edu.cn; yuhongbo@hust.edu.cn

M. He

State Key Laboratory of Intelligent Manufacturing Equipment and Technology, School of Mechanical Science and Engineering, Huazhong University of Science and Technology, Wuhan, 430074, China

J. Wang

Wuhan Second Ship Design and Research Institute, Wuhan, 430205, China

Y. Zhou

Guangxi Shenguan Collagen Technology Research Institute, Guangxi Shenguan Collagen Biological Group, Wuzhou, 543000, China

Correspondence to: xiasheng@vip.163.com

^#^ These authors contributed equally

**Contents**

Supplementary figures 1-14

Supplementary notes 1-5

Supplementary tables 1

Supplementary references 1-39

**Figure S1.** a) SEM image of collagen exhibiting a natural nanofibrous material at the micro-scale. b) SDS-PAGE analysis of bovine type I collagen (M: molecular marker; Col: collagen). Two distinct α chains (*α_1_* and *α_2_*), typical of type I collagen, along with their dimer, a *β* chain, were observed in the profile.

**Figure S2.** Schematic illustration of collagen-lignin composite materials formation process.

**Figure S3.** Fourier transform infrared (FTIR) spectra of a) S-CLS, b) I-CLS and c) L-CLS (I-CLS: insoluble lignin; S-CLS: soluble lignin; L-CLS: longlive lignin).

**Note S1: X-ray diffraction patterns analysis**

X-ray diffraction (XRD) analysis of S-CLSs (soluble lignin) was used to evaluate the influence of lignin content on the crystal structure of collagen fibers. As shown in Figure S4, the native collagen fibers show characteristic diffraction peaks at 2*θ* = 7.2°, 20.8° and 31.6°.^[1]^ The sharp peak at 2*θ* = 7.2° indicates the intermolecular lateral packing distance between the molecular collagen chains, and the peak intensity gradually decreased with the addition of lignin, suggesting that the distance between molecular chains was increased.^[2]^ The extensive broadening peak at 2*θ* = 20.8° corresponded to amorphous scattering resulting from unordered components of collagen fibers. All samples displayed similar XRD patterns with a slight increase in the intensity of the band for the sponges with lignin, which was indicative of the increase in the structural order.^[3]^ Furthermore, the narrow peak at 2*θ* = 31.6° corresponded to the unit height, which was typical of the triple helical structure. It was evident that the triple helix structure of the collagen nanofiber was maintained in all S-CLSs, in consistency with FTIR results.^[4]^

**Figure S4.** X-ray diffraction patterns (XRD) of S-CLSs (soluble lignin) with different lignin contents.

**Note S2: Influence of thickness on sound absorption performance**

Thickness is the most direct parameter to reflect the noise absorption property of the sponges. A series of studies were conducted on different thicknesses, ranging from 10 mm to 60 mm, to investigate how thickness affected the sound absorption performance of the CLSs. The results of CS and S-CLS (soluble lignin), shown in Figure S5a-b and Figure S5c-d respectively, indicated that as the thickness increased, the SAC of the sponge gradually increased and the absorption peak moved towards lower frequencies. For the S-CLSs, the NRC value (Figure S5d) increases significantly from 0.20 with a thickness of 10 mm to 0.74 with a thickness of 60 mm. The reason for this behavior lies in the physics, where the absorption capability of low-frequency noise improves as the thickness of the porous material increases, while other structural parameters stay the same. The longer propagation paths and more intricate channels inside the material are responsible for this phenomenon.^[5]^ Several studies have concluded that there is a direct relationship between the SAC and the thickness of the material being analyzed. Zong et al. ^[6]^ conducted a study on the sound absorption performance of flexible ceramic nanofibrous sponges (FCNSs) and analyzed their results based on the FCNSs’ thickness. Upon analyzing the data, it was found that an increase in thickness resulted in improved absorption coefficients and NRC values. Notably, the NRC values of FCNSs at thickness levels of 10, 20, 30 and 40 mm were found to be 0.1, 0.3, 0.4 and 0.5, respectively, which were lower than the values observed in our study. It is evident that enhancing the sound absorption capacity of fibrous materials can be achieved by increasing their thickness.^[7]^

However, in practical applications, it is not feasible to increase the thickness of porous materials infinitely due to cost and space limitations, particularly in the context of aerospace equipment.^[8]^ Additionally, the effective sound absorption coefficient of a porous absorber is accomplished when the absorber thickness is one-tenth of the incident sound wavelength.^[9]^ Therefore, the thickness of noise-absorbing materials generally varies from 10 to 40 mm, depending on the available installation space. In this study, further research was conducted using a material thickness of 30 mm.

**Figure S5.** The effect of thickness on the sound absorption properties. a) Sound absorption coefficient and b) noise reduction coefficient (NRC) of the collagen sponges with different thicknesses. c) Sound absorption coefficient and d) noise reduction coefficient of the S-CLSs (soluble lignin) with different thicknesses.

**Figure S6.** The effect of lignin content (insoluble lignin, IL) on the acoustic and mechanical properties. a) Sound absorption coefficient of CLSs with different IL contents. b) NRC of the sponges with different IL contents. c) Specific surface acoustic impedance of the sponges with different IL contents. d) Compressive stress-strain curves with different IL contents.

**Figure S7**. The effect of lignin content (longlive lignin, LL) on the acoustic and mechanical properties. a) Sound absorption coefficient of CLSs with different LL contents. b) NRC of the sponges with different LL contents. c) Specific surface acoustic impedance of the sponges with different LL contents. d) Compressive stress-strain curves with different LL contents.

**Figure S8**. Porosity of the sponges with (a) different lignin contents and (b) different layers (I-CLS: insoluble lignin; S-CLS: soluble lignin; L-CLS: longlive lignin).

**Note S3: Moisture insulation property analysis**

The insulation properties of moisture are essential for the practical application of sound absorption materials.^[10]^ The contact angle of water (WCA) of materials can reflect the moisture resistance property of materials toward a given liquid. The WCAs on the surface of the S-CLSs (soluble lignin) were investigated in Figure S9a. It was evident that as the lignin content increased, the value of WCA decreased from approximately 90° for the pure collagen sponge to approximately 60° for the collagen-lignin sponge with a 1% lignin loading. The possible reason could be divided into two parts. On the one hand, a large amount of hydroxyl groups within lignin was introduced into collagen side chains, which may improve the hydrophilicity of CLSs ^[11]^. On the other hand, the addition of lignin resulted in the porous sponges obtaining microscale structures and rougher surfaces, which means that the hydrophilicity of the sponges was promoted accordingly. Considering the aforementioned information, the hydrophobic method was adopted by wrapping a layer of polydimethylsiloxane (PDMS) onto the surface of the CLSs to achieve the intended effect.^[12]^ PDMS with a Si−O backbone possesses several advantages such as nontoxicity, super hydrophobicity, easy fabrication, chemical, and mechanical robustness and high flexibility.^[13]^ Therefore, it can compensate for the poor water resistance of both materials in this study. After being soaked and cured in the coating solution, the hydrophobic sponges (HCLSs) exhibited a high apparent water contact angle of 120°, which endows them with enhanced moisture insulation and maintains stable acoustic absorption properties. To assess the moisture resistance, we determined the weight retention rate by placing the HCLS in an ambient environment and weighing the mass at regular intervals. The sample weight remained remarkably consistent, with no discernible weight fluctuations (Figure S9b), indicating that the sponge does not absorb water from the air. The moisture resistance property of HCLS was further demonstrated by moisture absorption and desorption tests in a high-humidity environment (Figure S9c and Figure S9d). After exposing the HCLSs to water mist generated by a commercial humidifier for 12 hours and conducting periodic weight measurements, we observed a 55% weight increase during high-humidity exposure. Notably, when transferred to an ambient environment with 65% relative humidity, the adsorbed water in the samples was rapidly desorbed, allowing them to return to their original weight in approximately 2.5 hours (Figure S9e). This demonstrates that the sponges possess good moisture resistance properties. Water primarily accumulates on the surfaces of the fibers and membranes due to their hydrophobic properties, resulting in rapid moisture desorption.

**Figure S9.** a) Water contact angle (WCA) of S-CLSs (soluble lignin) with different lignin contents and the sponge with superhydrophobic treatment. The symbols denote the experimental points, and the solid line serves to guide the eye. b) Weight retention rate of the HCLS kept in ambient environment for different periods of time. c) Optical image of the setup for the moisture resistance property determination at a high-humidity atmosphere. d) Optical image of the HCLS in the water mist produced by a commercial humidifier. e) Moisture absorption and desorption properties of the HCLS.

**Note S4: Thermogravimetric analysis of CLSs**

Thermogravimetric analysis curves of CLSs with different lignin contents are shown in Figure S10. As complete degradation of collagen and lignin was done at 700 °C, TGA was performed up to 1000 °C to maximally degrade the samples.^[14, 15]^ The weight loss of the CLSs mainly occurred in three stages: 30–120 °C, 250–450 °C and 400–1000 °C. The first stage with small weight loss was related to the evaporation of free water and bound water.^[16]^ The group with added lignin will have a higher weight loss of water mass, and this phenomenon illustrates that the addition of lignin increases the hygroscopic properties of the sponges. The second stage of weight loss was observed between 250 °C and 450 °C, attributed to the polymer decomposition of collagen and lignin, and then continuous weight loss up to 1000 °C was observed. During the second step of degradation, CLSs containing 0.05%, 0.1%, 0.5% and 1% lignin showed weight losses of almost 52.0%, 43.7%, 38.7% and 32.9%, respectively, which were lower than that of Col sponges (53.6%). It can be inferred that lignin has slowed down the decomposition rate of CLSs in the temperature range between 250 and 450 °C. After final thermal decomposition process, 28.6, 30.7, 36.2, 41.5 and 42.7% residues were left separately for CLSs consisting of 0–1% lignin. This result indicated that the addition of lignin could significantly enhance the thermal stability of the porous sponges.^[17]^

**Figure S10.** Thermogravimetric analysis curves of S-CLSs (soluble lignin) with different lignin contents.

**Figure S11.** a) Ignition test comparison of collagen-lignin sponge (soluble lignin) with collagen sponge in an alcohol burner flame (~ 30 mm thick samples). b) Infrared thermal images of collagen-lignin sponge and collagen sponge at 0 seconds and 30 seconds after combustion. c) Infrared thermal images of collagen-lignin sponge (CLS) and collagen sponge (CS) at a platform temperature of 200 °C for one hour (left) and after removal from the thermal plate (right).

**Figure S12.** SEM images of the hierarchical pore structure of S-CLSs (soluble lignin) for different specimens (S-CLS0 / S-CLS05), layers (surface / medium / bottom) and magnifications (scales 0.5 mm / 250 μm) associated with their pore size distribution measurements.

**Figure S13.** Experimental measurements of sound absorption coefficient of S-CLS (soluble lignin) at normal (0°) and inverse (180°) incident angles.

**Figure S14.** a) The simulation curves derived from the JCAPL-limp model at various oblique incidences. b) NRC of the simulation curves at various oblique incidences.

**Table S1.** Areal density and noise reduction coefficient (NRC) of materials in references.

| **Materials** | **Areal density**  **(mg cm^-2^)** | **Noise reduction**  **coefficient (NRC)** | **References** |
| --- | --- | --- | --- |
| CLS | 52 | 0.63089 | our work |
| CS | 28 | 0.47 | our work |
| Kenaf | 300 | 0.50 | ^[18]^ |
| Wood fiber | 600 | 0.66 | ^[18]^ |
| Mineralized wood | 780 | 0.21 | ^[18]^ |
| Hemp | 150 | 0.39 | ^[18]^ |
| Coconut | 300 | 0.49 | ^[18]^ |
| Straw | 600 | 0.69 | ^[18]^ |
| Cardboard | 1610 | 0.48 | ^[18]^ |
| Sheep wool | 160 | 0.53 | ^[18]^ |
| Cork | 300 | 0.26 | ^[18]^ |
| Ramie | 1772 | 0.6 | ^[19]^ |
| Jute | 1644 | 0.65 | ^[19]^ |
| Kenaf | 74 | 0.11 | ^[20]^ |
| Jute | 73 | 0.13 | ^[20]^ |
| Coir fiber | 585 | 0.54 | ^[21]^ |
| Corn husk fiber | 600 | 0.53 | ^[22]^ |
| Yucca Gloriosa | 600 | 0.4 | ^[23]^ |
| Wool | 35 | 0.3 | ^[24]^ |
| Sugarcane bagasse waste fiber | 600 | 0.53 | ^[25]^ |
| Kenaf fiber | 600 | 0.52 | ^[26]^ |
| Date palm fiber | 600 | 0.4 | ^[27]^ |
| Shave and Coffee Silver Skin | 600 | 0.46 | ^[28]^ |
| Sisal fiber | 600 | 0.37 | ^[29]^ |
| Coconut husk fiber | 600 | 0.4 | ^[29]^ |
| Insulwood | 110 | 0.37 | ^[30]^ |
| Rice straw stem | 460 | 0.389 | ^[31]^ |
| Water Hyacinth Stems | 337 | 0.37 | ^[32]^ |
| Coarse wool | 410 | 0.23 | ^[33]^ |
| Tragacanth Gum / Persian gum | 35 | 0.44 | ^[34]^ |
| Pineapple aerogels | 97.89 | 0.52 | ^[35]^ |
| Softwood Kraft pulp | 200 | 0.19 | ^[36]^ |
| Bamboo | 600 | 0.68 | ^[37]^ |
| Commercial polyurethane | 158 | 0.4 | ^[38]^ |
| Commercial melamine foam | 27 | 0.3 | ^[39]^ |
| Commercial nonwoven felt | 53 | 0.43 | ^[39]^ |

**Note S5: Theory of thermal-viscous dissipation in porous media acoustics**

In the acoustic theory of porous media, the sound absorption induced by a porous structure is caused by the thermo-viscous dissipation between the solid medium (collagen-lignin network) and the fluid medium (air). The mechanism is divided into: (i) viscous friction loss and (ii) thermal conduction absorption. In the viscous friction loss, the thickness of the boundary layer between the fluid and the solid is calculated as $\delta_{v}=\sqrt{2\eta/(\omega\rho_{0})}$, which is related to the frequency of the acoustic wave. At low frequencies, the phenomenon can be calculated and derived directly from the Stokes equations, and thus to give the velocity field *v* and the pressure field *p*. However, at high frequencies, due to the decrease in thickness of the viscous boundary layer, the fluid behaves as an inertial state (no longer viscous). Then the velocity *v* derived from the gradient of the pressure *p* can be equivalently replaced by the electric field *E* derived from the gradient of the electric potential *ψ*, so that the Stokes equations can be approximated to the electric potential equations, see Table 1. On the other hand, for the thermal conductivity absorption, the temperature rise and fall (*ΔT*, denoted as *w*) of the air in the pore inside materials is due to the alternating changes in pressure *p*, and can be characterized by the Fourier's law of thermal conduction, which is eventually reduced to the form of Poisson's equation in Table 1. Therefore, these intermediate variables, such as the electric potential field *ψ* and the excess temperature field *w*, are needed for calculating the final absorption coefficient as a function of frequency.

Supplementary References

[1] Meng Z, Zheng X, Tang K, Liu J, Ma Z, Zhao Q, *Int J Biol Macromol*. **2012**, *51*, 440.

[2] Andonegi M, Irastorza A, Izeta A, de la Caba K, Guerrero P, *Pharmaceutics*. **2020**, *12*.

[3] Andonegi M, Heras KL, Santos-Vizcaino E, Igartua M, Hernandez RM, de la Caba K, Guerrero P, *Carbohydr Polym*. **2020**, *237*, 116159.

[4] Giraud-Guille M-M, Besseau L, Chopin C, Durand P, Herbage D, *Biomaterials*. **2000**, *21*, 899.

[5] Jorge P. Arenas; Malcolm J. Crocker, *Sound Vib*. **2010**, *44*, 12.

[6] Zong D, Cao L, Yin X, Si Y, Zhang S, Yu J, Ding B, *Nat Commun*. **2021**, *12*, 6599.

[7] Cao L, Fu Q, Si Y, Ding B, Yu J, *Compos Commun*. **2018**, *10*, 25.

[8] Zhu J, Sun J, Tang H, Wang J, Ao Q, Bao T, Song W, *Powder Technol*. **2016**, *301*, 1235.

[9] Kalauni K, Pawar SJ, *J Porous Mat*. **2019**, *26*, 1795.

[10] Nine MJ, Ayub M, Zander AC, Tran DNH, Cazzolato BS, Losic D, *Adv Funct Mater*. **2017**, *27*, 1703820.

[11] Yong M, Zhang Y, Sun S, Liu W, *J Membr Sci*. **2019**, *575*, 50.

[12] Zhang Y-Q, Jiang Y-H, Qin Y-N, An Q-D, Xiao L-P, Wang Z-H, Xiao Z-Y, Zhai S-R, *Colloids Surf A Physicochem Eng Asp*. **2022**, *643*, 128790.

[13] Tong H, Chen H, Zhao Y, Liu M, Cheng Y, Lu J, Tao Y, Du J, Wang H, *Colloids Surf A Physicochem Eng Asp*. **2022**, *648*, 129228.

[14] Iqbal B, Muhammad N, Jamal A, Ahmad P, Khan ZUH, Rahim A, Khan AS, Gonfa G, Iqbal J, Rehman IU, *J Mol Liq*. **2017**, *243*, 720.

[15] Zhang J, Fleury E, Chen Y, Brook MA, *RSC Adv*. **2015**, *5*, 103907.

[16] Zhang T, Yu Z, Ma Y, Chiou B-S, Liu F, Zhong F, *Food Hydrocoll*. **2022**, *124*, 107270.

[17] Zhao Z, Cannon FS, Nieto-Delgado C, *Carbon*. **2019**, *154*, 254.

[18] Berardi U, Iannace G, *Appl Acoust*. **2017**, *115*, 131.

[19] Yang W, Li Y, *Sci China Technol Sci*. **2012**, *55*, 2278.

[20] Na Y, Cho G, *Fibers Polym*. **2010**, *11*, 782.

[21] Taban E, Tajpoor A, Faridan M, Samaei SE, Beheshti MH, *Acoust Aust*. **2019**, *47*, 67.

[22] Fattahi M, Taban E, Soltani P, Berardi U, Khavanin A, Zaroushani V, *J Build Eng*. **2023**, *77*.

[23] Soltani P, Taban E, Faridan M, Samaei SE, Amininasab S, *Appl Acoust*. **2020**, *157*.

[24] Ballagh KO, *Appl. Acoust.* . **1996**, *48*, 101.

[25] Mehrzad S, Taban E, Soltani P, Samaei SE, Khavanin A, *Build Environ*. **2022**, *211*.

[26] Taban E, Soltani P, Berardi U, Putra A, Mousavi SM, Faridan M, Samaei SE, Khavanin A, *Build Environ*. **2020**, *180*.

[27] Taban E, Khavanin A, Ohadi A, Putra A, Jafari AJ, Faridan M, Soleimanian A, *Build Environ*. **2019**, *161*.

[28] Abdi DD, Monazzam M, Taban E, Putra A, Golbabaei F, Khadem M, *Appl Acoust*. **2021**, *182*.

[29] Silva CCBd, Terashima FJH, Barbieri N, Lima KFd, *Appl Acoust*. **2019**, *156*, 92.

[30] Zhao X, Liu Y, Zhao L, Yazdkhasti A, Mao Y, Siciliano AP, Dai J, Jing S, Xie H, Li Z, He S, Clifford BC, Li J, Chen GS, Wang EQ, Desjarlais A, Saloni D, Yu M, Kośny J, Zhu JY, Gong A, Hu L, *Nat Sustain*. **2023**, *6*, 306.

[31] Kolya H, Kang C-W, *Clean Technol Envir*. **2023**, *25*, 3219.

[32] Olivares-Marín M, Román S, Gómez Escobar V, Moreno González C, Chaves-Zapata A, Ledesma B, *J Clean Prod*. **2023**, *425*.

[33] Broda J, Kobiela-Mendrek K, Bączek M, Rom M, Espelien I, *J Nat Fibers*. **2023**, *20*.

[34] Khorami M, Rangkooy H, Dehaghi BF, Salimi A, *J Polym Environ*. **2023**.

[35] Do NHN, Luu TP, Thai QB, Le DK, Chau NDQ, Nguyen ST, Le PK, Phan-Thien N, Duong HM, *Mater Chem Phys*. **2020**, *242*.

[36] Ayub M, Nor MJM, Fouladi MH, Zulkifli R, Amin N, *Acoust Phys*. **2012**, *58*, 246.

[37] T. Koizumi NT, A. Adachi, *High Performance Structures and Materials*. **2002**, *59*, 157.

[38] Witthayolankowit K, Boonyarit J, Srichola P, Rungruangkitkrai N, Apipatpapha T, Chollakup R, *J Nat Fibers*. **2023**, *20*.

[39] Cao L, Si Y, Wu Y, Wang X, Yu J, Ding B, *Nanoscale*. **2019**, *11*, 2289.
